# Supplementary material for: Partial label learning for automated classification of single-cell transcriptomic profiles
Source: PLoS Comput Biol. 2024 Apr 5;20(4):e1012006. doi: 10.1371/journal.pcbi.1012006 (PMC11023635; doi:10.1371/journal.pcbi.1012006)
Supplement: S1 Text — (PDF) [file pcbi.1012006.s001.pdf]

S1 Text. Supplementary Methods

We focus in this section on the implementation details of the methods compared in the paper. More specifically we provide details on the methods that are optimized with the Iterative Refinement Learning (*IRL*) Algorithm (Algorithm 1 in section **Methods** of the main manuscript), and for methods whose extension to hierarchical classification has been investigated. We also provide details on variants of these methods when applicable, in particular, nonlinear extensions of the SVM-based and the Prototype-based approaches. All along this section we build on existing methods and mention when we detail a method which was not proposed before.

We do not provide any specific details for classification methods (Random Forest, Gradient Boosting and kernel SVM), whose extension to the *partially labeled* setting is implemented through the use of the Iterative Full Retraining (*IFR*) algorithm (Algorithm 2, described in the section **Methods** of the main manuscript) as this extension is straightforwardly implemented using their well known supervised learning strategy.

In the following, we use the same notations as in the main manuscript. Supervised learning is performed over a fully labeled (i.e. every training sample is associated to its true label) dataset $D = D_s = \{(x_i, y_i) \in \mathcal{X} \times \mathcal{Y}\}_{1 \leq i \leq l}$. To ease the reading, the partially labeled extension is detailed for the case where the training dataset includes partially labeled samples only, $D = D_{pl} = \{(x_i, Y_i) \in \mathcal{X} \times P(\mathcal{Y})\}_{1 \leq i \leq m}$, but extension to a mixed case with both supervised and partially labeled samples is straightforward. Finally, we discuss a hierarchical variant of the methods, that take into account the knowledge of a hierarchical organization of the labels.

Note that whenever it was possible, we used the state-of-the-art implementation for machine learning methods from scikit-learn [1].

In the following, we suppose that our dataset is $\mathcal{X} = \mathbb{R}^d$, where d represents the number of features and the set of labels $\mathcal{Y}$ is composed of c labels.

Multiclass SVM

We first present the supervised case where we propose to use standard multiclass SVM. We then present variants dedicated to partially labeled data, its extension to hierarchical classification, and a nonlinear version of these. As stated above any mix of these ideas may be easily implemented (e.g. hierarchical classification with both supervised and partially labeled data).

Supervised setting. A multiclass linear SVM is defined through a weight matrix $W \in \mathbb{R}^{d \times c}$ which is composed of c column vectors $\{w_j\}_{1 \leq j \leq c} \in \mathbb{R}^d$ which stand for each label's classifier weights. We note f_W the SVM model with parameters W. One defines a score for a sample and a label γ as $score(f_W, x, \gamma) = \langle w_\gamma, x \rangle$. A sample x is classified according to $\arg\max_\gamma score(f_W, x, \gamma) = \arg\max_\gamma \langle w_\gamma, x \rangle$. Hence a sample x whose class is y is correctly classified if $\arg\max_\gamma \langle w_\gamma, x \rangle = y$.

The supervised learning of a multiclass SVM on a dataset D_s may be cast as the following optimization problem:

$$\min_{W,\xi} \frac{1}{2}||W||^2 + \frac{\mu}{l} \sum_i^l \xi_i \tag{1}$$

$$\text{such that } \forall i, \langle w_{y_i}, x_i \rangle - \max_{\gamma \neq y_i} \langle w_\gamma, x_i \rangle \geq 1 - \xi_i \tag{2}$$

$$\text{and } \forall i, \xi_i > 0 \tag{3}$$

where ξ_i are slack variables and μ is a hyperparameter that allows to tune the tradeoff between the two terms in the loss function. Note that there are as many constraints as the number of training samples. Each constraint encodes that the ith sample is correctly satisfied with a margin, with slack variables enabling some errors. This problem can be solved by gradient descent by minimizing its unconstrained form which we put in the same shape as in Eq. (2) of the main manuscript:

$$\min_{W,\xi} \frac{1}{2}||W||^2 + \frac{\mu}{l} \sum_i^l loss(f_W, x_i, y_i) \tag{4}$$

$$= \min_{W,\xi} \frac{1}{2}||W||^2 + \frac{\mu}{l} \sum_i^l \max\left(0, 1 - [\langle w_{y_i}, x_i \rangle - \max_{\gamma \neq y_i} \langle w_\gamma, x_i \rangle]\right) \tag{5}$$

Partial labeling setting. The extension of SVM to the partial labeling setting has already been investigated, we take inspiration from the method in [2]. In the pure partial label learning setting, the training set is $D = D_{pl} = \{(x_i, Y_i), s.t. Y_i \subset \mathcal{Y}\}_{1 \leq i \leq m}$ and the learning is cast as the following minimization problem:

$$\min_{W,\xi} \frac{1}{2}||W||^2 + \frac{\lambda}{m} \sum_{i}^{m} \min_{\tilde{y}_i \in Y_i} loss(f_W, x_i, \tilde{y}_i) \tag{6}$$

$$= \min_{W,\xi} \frac{1}{2}||W||^2 + \frac{\lambda}{m} \sum_{i}^{m} \min_{\tilde{y}_i \in Y_i} \max\left(0, 1 - [\langle w_{\tilde{y}_i}, x_i \rangle - \max_{\gamma \notin Y_i} \langle w_\gamma, x_i \rangle]\right) \tag{7}$$

This formulation follows the form of Eq. (3) in the main manuscript, where $\tilde{y}_i \in Y_i$ stands for the guessed label for a partially labeled training sample $(x_i, Y_i) \in D_{pl}$ and $\lambda > 0$ tunes the tradeoff between the two terms of the objective function.

Both algorithms (IRL and IFR) may be used to optimize this objective where the optimization algorithm iterates inferring new guess for $\tilde{y}_i$ and re-estimate the model's parameters W (through refinement or full retraining).

Hierarchical variant. A classic way of exploiting the hierarchical organization of labels is to consider that it is a more severe error if a predicted label is very far in the label hierarchy from the true label than if it is closer. This may be integrated into an SVM formulation with the slack scaling strategy inspired by the structured output prediction field [3]. The related optimization problem becomes the following where the margin $+1$ is replaced by a quantity that is related to the distance between the labels, we use here $C_{y_i,\gamma}$. The more distant a label γ is from the true label y_i in the hierarchy, the larger the difference between the scores for these two labels should be. Note that in this case the objective function is an upper bound of the average distance from the predicted label to the true one whereas the loss function of standard multiclass SVM is an upper bound of the number of classification errors. Of course this hierarchical classification strategy may be combined with the partial labeling approach.

$$\min_{W,\xi} \frac{1}{2}||W||^2 + \frac{\mu}{l} \sum_{i}^{l} \max\left(0, C_{y_i,\gamma} - [\langle w_{y_i}, x_i \rangle - \max_{\gamma \neq y_i} \langle y_\gamma, x_i \rangle]\right) \tag{8}$$

Nonlinear extension The linear SVM-like formalization above (that we name *SVM* in our experiments) yields a natural nonlinear extension with the kernel trick. We performed experiments with such a usual nonlinear implementation of SVM, that we call k-SVC in our experiments. It is trained with the IFR algorithm and, hence, does not scale well to large datasets. To enable efficient implementation and scaling to large datasets as well as to ease the extension to the hierarchical classification setting, we also considered using the *IRL* algorithm (Algortihm 1) and relied on a kernel approximation with a Radial Basis Function approach [4], where the score for a class y and for a sample x is implemented by $\langle w_y, \tilde{\psi}(x)\rangle$ with $\tilde{\psi}$ an appropriately chosen mapping function. It is a rather standard implementation, and we used the one provided by the scikit-learn package [1]. We name $\tilde{k}$-SVM this implementation of SVM.

Prototype-based approach

We first introduce a prototype-based approach that is inspired by works in extreme classification (i.e. when the number of classes is large) [5]. We consider first the supervised setting, then the partially labeled setting, and discuss variants, such as a nonlinear version and an extension incorporating the hierarchical structure of the label set.

Supervised setting. We build on the method in [5] as our starting point. It consists in projecting simultaneously data samples and one-hot representation of the labels in a common representation space $\mathcal{Y}$ where a prototype-based approach is used to classify data samples (i.e. a data sample is classified with the label whose projection is closest to the projection of the sample). The projection is learned to maximize a margin criterion. While the method has been designed for extreme classification, i.e. hierarchical classification when the number of labels is large (hundreds of thousands of labels or more), we exploit here a simplified version of the method. A projection operator $P \in \mathbb{R}^{d\times c}$ is learned to project data samples from $\mathcal{X}$ to $\mathbb{R}^c$, with c being the number of labels where a nearest neighbor classification rule is applied. The projections of the labels in $\mathbb{R}^c$ remain fixed and are set to either a one-hot-encoding or a multi-dimensional scaling projection (MDS) [6], the best projection is set by cross-validation. The final encoding of label is given by a matrix $\phi = \{\phi_i\}_{1\leq i\leq c}$, composed of c columns vectors of dimension c.

To frame the approach similarly to our generic formalization in the Methods section of the paper, we note f_P the prototype-based mode, $f_P(x)$ the prediction for a sample x, and $score(f_P, x, y) = -||Px - \phi_y||_2^2$ the score for model f_P, input sample x and output label y. Note that the score is set minus the norm to be consistent with the maximization strategy of the score in the main manuscript. The learning is cast as the following optimization problem:

$$\min_P \frac{1}{2}||P||^2 + \frac{\mu}{l}\sum_i^l loss(f_P, x_i, y_i) \tag{9}$$

$$= \min_P \frac{1}{2}||P||^2 + \frac{\mu}{l}\sum_i^l \max_{\gamma \neq y_i}\Big(0, 1 - \Big[||Px_i - \phi_{y_i}||_2^2 - ||Px_i - \phi_\gamma||_2^2\Big]\Big) \tag{10}$$

The above objective function may be optimized with both algorithms *IRL* and *IFR*, similarly to the SVM above. Note that to achieve better results we investigated optimizing a combined objective by adding a regression term to the objective function $L_{reg}(P) = \frac{\nu}{l}\sum_i^l ||Px_i - \phi_{y_i}||^2$ where $\nu > 0$ is a hyper-parameter to set with cross-validation.

Partially labeled setting and hierarchical variant. Like for SVM-based methods, one may extend the supervised method to the partially labeled setting by introducing latent variables. Noting the labeling $\tilde{y}_i \in Y_i$ of samples $(x_i, Y_i) \in D_{pl}$, the learning of the prediction model may then be cast as the following optimization problem, where one may add a regression term to the loss, in a similar way to the previous section. :

$$\min_P \frac{1}{2}||P||^2 + \frac{\lambda}{m}\sum_i^m \min_{\tilde{y}_i \in Y_i} \max_{\gamma \notin Y_i}\Big(0, 1 - \Big[||Px_i - \phi_{\tilde{y}_i}||_2^2 - ||Px_i - \phi_\gamma||_2^2\Big]\Big) \tag{11}$$

Again we propose to learn the prototype-based model in the partial labeling setting by alternating the inference of latent variables $\tilde{y}_i$ based on the current model's parameters, and the re-estimation of the model's parameters through supervised learning.

Finally, the hierarchical variant may again be derived by introducing margin scaling replacing the 1 margin in Eq. (10) and (11) by distances $C_{y_i,\gamma}$ between two labels y_i and γ.

Nonlinear extension. In order to investigate nonlinear projection, one may replace $P \in \mathbb{R}^{d \times c}$, by a Neural Network. We looked at different architectures and found that using 2 hidden layers, whose number of neurons is set to the number of labels and with hyperbolic tangent activation function, was a well-adapted architecture to our problem. More complex models were often subject to overfitting because of the small amount of training data. In our experiments we name *PB-l* the Prototype-based method exploiting a linear projection operator and *PB-nn* the Prototype-based method with a nonlinear neural network projection operator

Euclidean vs Correlation distance. We explored different metrics and replaced the Euclidean distance with the correlation distance, which has been shown to be relevant for transcriptomic data [7]. In this *Correlation distance* variant, all Euclidean distances in the above equations are replaced by:

$$d_{Correl}(x_1, x_2) = 1 - \frac{\sum_{g=1}^{p} (x_{1g} - \bar{x}_1)(x_{2g} - \bar{x}_2)}{\|x_1 - \bar{x}_1)\| \times \|x_2 - \bar{x}_2\|}$$

where $\bar{x}_1$ stands for the mean value among all the d features of x_1.

k-NN

We build here on standard k-NN [8] and on a variant of k-NN for partially labeled data that has been proposed in [9]. We propose an extension to hierarchical classification.

Supervised setting. The classification of a test sample is performed in two steps: first, find the k_{nn} nearest neighbors of the sample to be classified in the training dataset, then select the most frequent label within these neighbors. Noting $top_{k_{nn}}(x)$ the set of the k_{nn} nearest neighbors of an example x in the training set D_s, the k-NN classifier decision rule is defined as:

$$\hat{y} = \arg\max_{\gamma \in \mathcal{Y}} \sum_{x_j \in top_{k_{nn}}(x)} \mathbb{1}_{y_j = \gamma} \text{ where } \mathbb{1}_{y_j=\gamma} = \begin{cases} 1 \text{ if } y_j = \gamma \\ 0 \text{ else} \end{cases} \quad (12)$$

Partial labeling setting. We used a variant proposed in [9] to handle a training dataset $D = D_{pl} = \{x_i, Y_i, s.t. Y_i \in \mathcal{P}(\mathcal{Y})\}$. The decision rule is the following:

$$\hat{y} = \arg\max_{\gamma \in \mathcal{Y}} \sum_{x_j \in top_{k_{nn}}(x)} \sum_{y \in Y_j} \mathbb{1}_{y=\gamma} \tag{13}$$

It is a straightforward extension of k-NN to this setting where all labels in the labeling sets of the k_{nn} nearest neighbors count equally, with a majority vote at the end to determine the class of an incoming sample.

Hierarchical variant. We designed a hierarchical variant of k-NN by relying again on the matrix C, which may be applied in the fully supervised setting and in the partial label setting. The idea is that all the labels in all label sets of the nearest neighbors contribute but with a weight that depends on the distance in the hierarchy, we relied on the weights encoded in the matrix C and used the following decision rule :

$$\hat{y} = \arg\max_{\gamma \in \mathcal{Y}} \sum_{x_j \in top_{k_{nn}}(x)} \sum_{y \in Y_j} \frac{1}{2}^{C_{\gamma,y}} \tag{14}$$

where $C_{\gamma,y}$ is the dissimilarity between the labels γ and y.

References

[1] F. Pedregosa et al. "Scikit-learn: Machine Learning in Python". In: *Journal of Machine Learning Research* 12 (2011), pp. 2825–2830.

[2] Nam Nguyen and Rich Caruana. "Classification with partial labels". In: *Proceedings of the 14th ACM SIGKDD international conference on Knowledge discovery and data mining*. 2008, pp. 551–559.

[3] Ioannis Tsochantaridis et al. "Large Margin Methods for Structured and Interdependent Output Variables". In: *J. Mach. Learn. Res.* 6 (2005), pp. 1453–1484. URL: http://jmlr.org/papers/v6/tsochantaridis05a.html.

[4] Ali Rahimi and Benjamin Recht. "Random Features for Large-Scale Kernel Machines". In: *Advances in Neural Information Processing Systems*. Ed. by J. Platt et al. Vol. 20. Curran Associates, Inc., 2007. URL: https://proceedings.neurips.cc/paper_files/paper/2007/file/013a006f03dbc5392effeb8f18fda755-Paper.pdf.

[5] Chapelle Weinberger. "Large Margin Taxonomy Embedding with an Application to Document Categorization". In: *NeurIPS* (2008).

[6] Ingwer Borg and Patrick JF Groenen. *Modern multidimensional scaling: Theory and applications*. Springer Science & Business Media, 2005.

[7] Ebony Rose Watson et al. "How does the structure of data impact cell–cell similarity? Evaluating how structural properties influence the performance of proximity metrics in single cell RNA-seq data". In: *Briefings in Bioinformatics* 23.6 (Sept. 2022), bbac387. ISSN: 1477-4054. DOI: 10.1093/bib/bbac387. eprint: https://academic.oup.com/bib/article-pdf/23/6/bbac387/47143790/bbac387.pdf. URL: https://doi.org/10.1093/bib/bbac387.

[8] Belur V. Dasarathy. "Nearest neighbor (NN) norms: NN pattern classification techniques". In: 1991.

[9] Eyke Hüllermeier and Jürgen Beringer. "Learning from ambiguously labeled examples". In: *Intelligent Data Analysis* 10.5 (2006), pp. 419–439.
