# Supplementary material for: Partial label learning for automated classification of single-cell transcriptomic profiles
Source: PLoS Comput Biol. 2024 Apr 5;20(4):e1012006. doi: 10.1371/journal.pcbi.1012006 (PMC11023635; doi:10.1371/journal.pcbi.1012006)
Supplement: S2 Text — Comparison between IRL and IFR algorithms and additional results on kernel SVM. (PDF) [file pcbi.1012006.s002.pdf]

S2 Text. Supplementary Results

1. Description of the methods compared in the supplementary

We provide additional experimental results that investigate, first the respective merits of the two algorithms that we introduced in the paper, namely the *Iterative Refinement Learning (IRL)* (Algorithm 1) and the *Iterative Full Retraining (IFR)* (Algorithm 2); secondly the impact of the SVM implementation in the nonlinear case.

In the following Tab S1, we provide additional details for all the methods implemented in the main manuscript or in this supplementary results.

Methods	Acronym	Algo.	Implementation
Prototype Based with<br>- linear projection<br>- nonlinear projection (neural network)	 PB-l<br><br>PB-nn	 *IRL*<br><br>*IRL*	 Pytorch [1]<br><br>Pytorch [1]
Support Vector Machine<br>- linear<br>- nonlinear with kernel approximation	 SVM<br><br>$\hat{k}$-SVM	 *IRL*<br><br>*IRL*	 Pytorch [1]<br>Scikit-learn [2] ,<br>Pytorch [1]
Logistic Regression	LR	*IRL*	Pytorch [1]
k-Nearest Neighbors	kNN	N.A	Pytorch[1]
Random Forest	RF	*IFR*	Scikit-learn[2]
Extreme Gradient Boosting Method	XGBM	*IFR*	XGBM[3]
linear SVM with algorithm *IFR*	SVM-*IFR*	*IFR*	Pytorch[1]
kernel SVM	k-SVC	*IFR*	Scikit-learn[2]
LR with algorithm *IFR*	LR-*IFR*	*IFR*	Pytorch[1]

Table S1. Details of methods' acronyms, learning algorithm and implementation. *IRL* stands for Iterative Refinement Learning algorithm, *IFR* Iterative Full Retraining algorithm, *N.A* means not applicable.

Note that all the results reported in this supplementary material have been obtained with the same experimental protocol as results that are reported in the main manuscript, see Section **Datasets and experimental settings**, subsection **Experiments**. This means in particular that results are averaged over 5 train/test splits, after a 5-fold cross-validation grid-search.

2. Comparison of Algorithm IRL and IFR

We now compare the two learning algorithms that we described in detail in the main paper, namely the Iterative Refinement algorithm (*IRL*, Algorithm 1) and the Iterative Full Retraining algorithm (*IFR*, Algorithm 2), which we described in the **Methods** section of the manuscript. Recall that the difference lies in the optimisation scheme. Given a best candidate in the partial labeling at a time step t, *IRL* performs a single-step gradient re-estimation and then re-estimates the best candidates in the partial labeling; whereas the *IFR* algorithm performs a full training from scratch with the current affected candidates in the partial labeling and the next affectation of best candidates is performed after convergence.

Partially labeled setting To make a fair comparison between the two algorithms, we ran the same method (Logistic Regression and linear SVM, both trained with gradient descent optimization) but optimized with the two different algorithms. The results reported in Fig. S1 show that the *IRL* algorithm significantly outperforms the *IFR* algorithm whatever the dataset and the experimental setting for SVM implementations, while it is most often the opposite for LR models. We do not have any clear explanation for this behavior given that linear SVM and logistic regression are usually considered very similar classifiers. We hypothesize that tuning the hyperparameters of the logistic regression model may be more sensitive and would have required a larger grid search, but we limited the gridsearch effort to a similar budget whatever the method to gain a more fair comparison. This motivated us to systematically report the SVM results obtained with the *IRL* algorithm in the experimental study reported in the main manuscript.

Finally, we observe that for ensemble models, the more expressive the models are, the lower their accuracy on partially labeled data. In particular, for both RF and XGBM, we noticed an improved accuracy on partially labeled data when reducing the capacity of the model (e.g. lowering the maximal depth of the trees), which seems consistent with the idea that too much expressivity of a classifier, when learned with the *IFR* algorithm, would make it able to learn the first (possibly random) labeling of partially labeled data and hence suffer from strong overtraining.

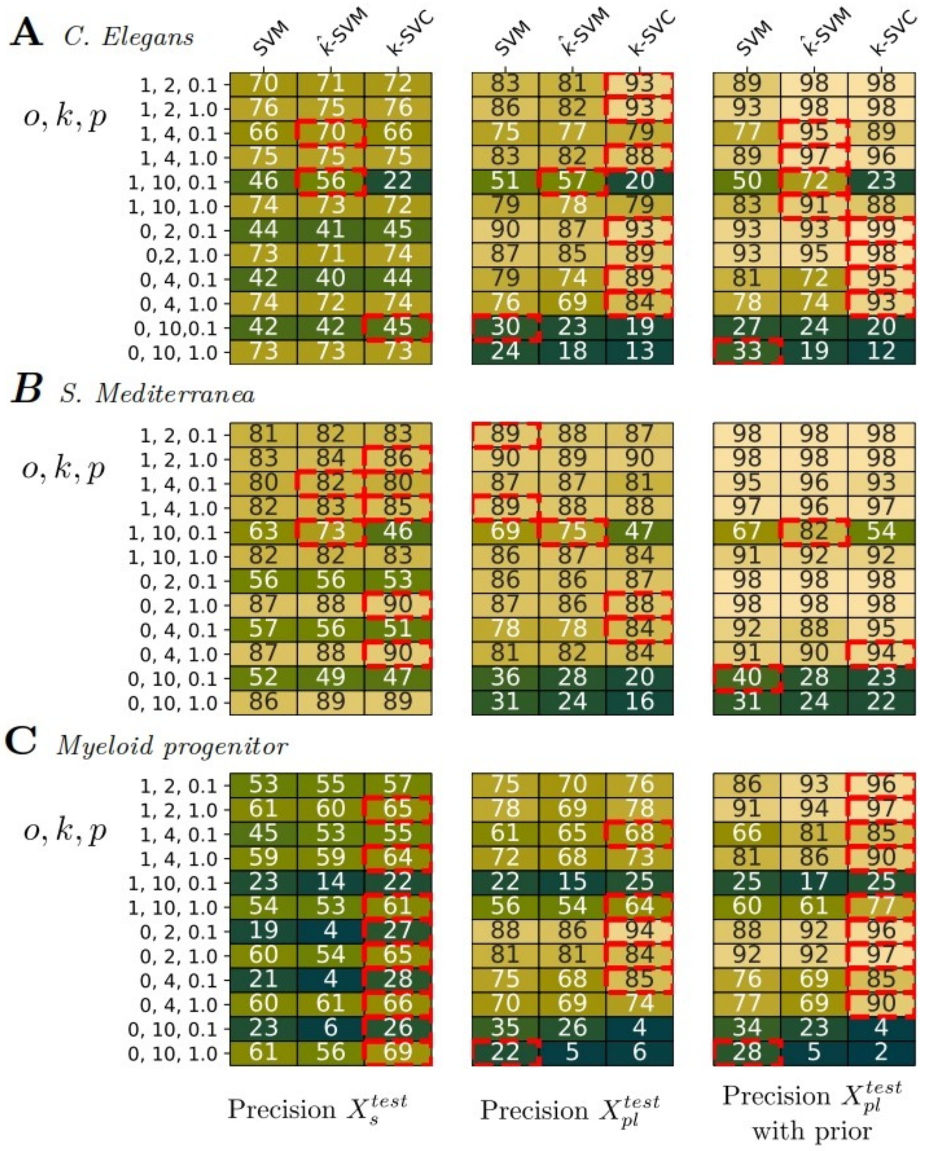

Fig S1. Comparison of *IRL* and *IFR* algorithm in the partially labeled scenarios.

A-B-C) Accuracies (in percentage) on the 3 real world datasets, where each heatmap corresponds to performance on X_s^{test}, Precision on X_{pl}^{test}, Precision on X_{pl}^{test} when prior information is available for the test too. Each row corresponds to the setting in partial label scenarios, depending on, by order, o: Overlap between supervised and partial label, k: number of partial label set, p: proportion of fully supervised training data. Each column corresponds to the performance of a method, where SVM stands for SVM with Algo *IRL* (as in the main manuscript), and SVM -*IFR* for SVM with Algo *IFR*. The red squares indicate the best performing method per row, i.e. per experimental setting, using a t-test and as significance criteria p-value < 0.1. We also compute paired t-test and highlight the results when model with *IRL* algorithm is performing significantly better than the *IFR* algorithm, we highlighted in magenta and in blue for the opposite situation.

3. SVM implementation and kernel approximation

As mentioned in S1 Text, we implemented nonlinear SVM in several ways to deal with the particular settings encountered in our experimental study. In particular, some datasets that we consider are quite large (e.g. all our artificial datasets include hundreds of thousands of training samples) preventing the use of standard implementation of kernel SVM, named here k-SVC (whose optimization does not scale with the number of training samples). Instead, we relied on the approximation of the Radial Basis Function (RBF) kernel through the Fourrier transformation [4] . This choice enables gradient descent optimization and scales very well with large datasets, we call this method $\hat{k}$-SVM.

Supervised results We first provide in Fig S2 a comparison of all the methods under investigation in a supervised setting. Performances (accuracy) are reported for the three real datasets and two sizes of the training set. One may see here that linear SVM and nonlinear SVM are similar in all these supervised classification tasks, and that the two nonlinear variants ($\hat{k}$-SVM and k-SVC) exhibit the same level of performance as the linear variant (SVM). More generally one sees that these models are more or less similar with well-performing baselines.

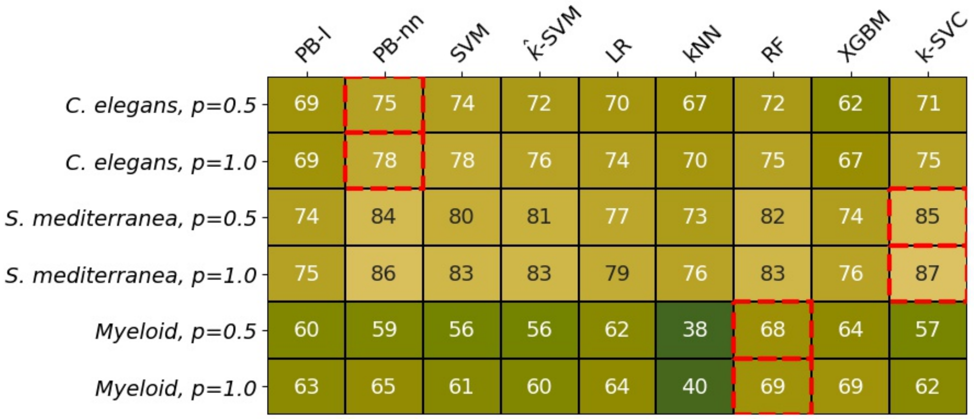

Fig S2. Comparison of classification performance of main baseline methods on the three real-world datasets in the fully supervised setting.

Performances (accuracy in %) are reported for two sizes of training datasets (full training dataset $p = 1.0$ or half of it, $p = 0.5$).

Partially labeled results Moreover, Fig S3 provides additional results of SVM implementations using both learning algorithms. In this figure, we compare the linear SVM and the nonlinear $\hat{k}$-SVM, both trained with the *IRL* algorithm, to standard kernel SVM trained with *IFR* algorithm, k-SVC. Compared to the results shown in Fig S1 we report results gained in the partial labeling setting so that one observes both the effect of the implementation and the training algorithm in these results. One sees here again that nonlinear SVM relying on a kernel approximation and true kernel SVM (k-SVC) are most often as good as each other, whatever the dataset

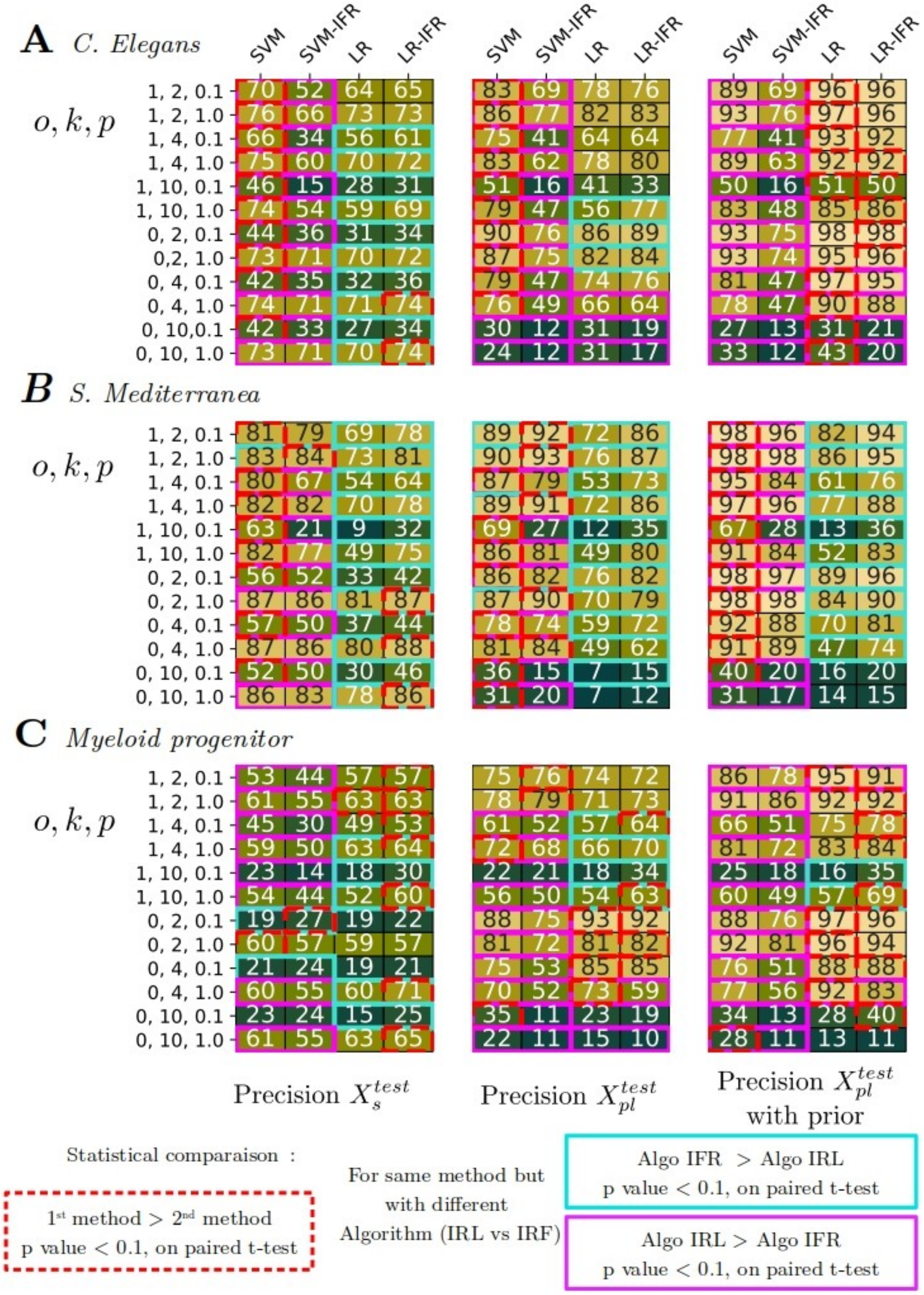

Fig S3. Comparison of SVM implementations in the partially labeled setting.

A-B-C) Accuracies (in percentage) on the 3 real-world datasets, where each heatmap corresponds to the Precision on X_s^{test}, Precision on X_{pl}^{test}, Precision on X_{pl}^{test} when prior information is available for test too. Each row corresponds to the setting in partial label scenarios, depending on, by order, o: Overlap between supervised and partial label, k: number of partial label set, p: proportion of fully supervised training data. Each column corresponds to the performance of a method, where SVM stands for linear SVM, $\hat{k}$-SVM for SVM with kernel approximation. Both of these implementations rely on Algo *IRL* (as in the main manuscript). Finally, kSVC stands for real kernel and its computation relies on *IFR* algorithm. The red squares indicate the best performing method per row, i.e. per experimental setting, using a t-test and as significance criteria p-value < 0.1.

and the setting, but with some exceptions where true kernel SVM (k-SVC) may significantly outperform $\hat{k}$-SVM. Yet we chose to systematically report

the performances of $\hat{k}$-SVM in the manuscript since only this implementation scales to all our datasets. Finally, note that linear SVM (SVM) are most often on par with their nonlinear counterparts.

References

[1] Adam Paszke et al. "PyTorch: An Imperative Style, High-Performance Deep Learning Library". In: *Advances in Neural Information Processing Systems 32*. Ed. by H. Wallach et al. Curran Associates, Inc., 2019, pp. 8024–8035. URL: http://papers.neurips.cc/paper/9015-pytorch-an-imperative-style-high-performance-deep-learning-library.pdf.

[2] F. Pedregosa et al. "Scikit-learn: Machine Learning in Python". In: *Journal of Machine Learning Research* 12 (2011), pp. 2825–2830.

[3] Tianqi Chen and Carlos Guestrin. "Xgboost: A scalable tree boosting system". In: *Proceedings of the 22nd acm sigkdd international conference on knowledge discovery and data mining*. 2016, pp. 785–794.

[4] Ali Rahimi and Benjamin Recht. "Random features for large-scale kernel machines". In: *Advances in neural information processing systems* 20 (2007).
