# Supplementary material for: Partial label learning for automated classification of single-cell transcriptomic profiles
Source: PLoS Comput Biol. 2024 Apr 5;20(4):e1012006. doi: 10.1371/journal.pcbi.1012006 (PMC11023635; doi:10.1371/journal.pcbi.1012006)
Supplement: Attachment — Submitted filename: Response.pdf [file pcbi.1012006.s003.pdf]

1 Summary of the changes

We thank the Reviewers for their careful reading of our manuscript and their positive assessment of our work. First of all, here is the list of the main changes that we made in our new version of the manuscript.

- We changed the whole **Materials and methods** section in the main manuscript to simplify its reading. The generic part has been rewritten and is kept in the main paper while details about methods, (the original **Materials and methods** section) have been moved to the supplementary material.
- To enable the use of new classification methods in the partial label learning setting (see below), particularly ensemble methods, we propose and investigate a dedicated learning strategy since the one we proposed originally is not well suited for such methods.
- We updated the Results section to include experimental results obtained with a few new methods for a more extensive comparison: Logistic Regression on all the datasets, and ensemble methods (Random Forest and Extreme Gradient Boosting Method) for the 3 real-world datasets since these latter methods do not scale well to our larger artificial datasets.
- We submit two supplementary files: the first one, called **S1 Text**, contains details on the methods. The second one called **S2 Text**, provides additional results to compare the various implementations of SVM methods and to compare the original and the new learning strategies we investigated for solving the partially labeled problem.
- In addition to the answers we write here, we provide a file, **Diff.pdf**, that highlights the changes between the two submission rounds. Note that we do not make the changes in section **Materials and methods** appear, as this section has been fully rewritten and the diff file would be hard to follow.
- We finally updated our GitHub repository to include the added methods under investigation (LR, RF, XGBM, kernel SVC): https://github.com/paulvill/plRNAseq.

Additional changes

- Correcting errors: We made some corrections on some performances but these changes do not affect the main messages in the discussion. In Fig 8 of the main manuscript, we identified erroneous numbers that came from an implementation error in the prediction stage of flat kNN with prior information. Moreover, we have corrected the values of some performances which were quoted in the discussion and which were different from those reported in the figures (lines 424 and 670 in the main manuscript).

- Improved writing: We have made some editorial changes to the text. We replaced *non linear* by *non-linear*, *single cell* by *single-cell* etc. We also systematically replaced the multiple namings that we used previously *weakly supervision* and *partial label*, by *partially labeled*. We changed notations accordingly and replaced X_{ws} by X_{pl}. We also changed the notation B to D which stands for Dataset. We also changed the acronym *k-NN* to *kNN* to avoid confusion with the *nn* suffix in *PB-nn*, which stands for neural network. Finally, to make things clearer we added a table that summarizes methods and acronyms in the main manuscript (Table 1 and **S2 Text**).

2 Point-by-point response

In what follows we take up the reviewers' comments one by one and respond to them by pointing out the changes made to the document.

Reviewer #1: *Senoussi et al. have performed the partial label single cell annotation to overcome the herculean task of specifying the labels for each cell. They have used different machine learning (Prototype based, SVM, and KNN) methods for partial automated single cell labeling on simulated and real-world datasets. They identified that non-linear prototype based works best both in simulated and real-world dataset and had a comparable accuracy with the fully supervised dataset. The manuscript has been well written and analyzed well.*

Authors: Thank you very much for this positive comment.

Reviewer #1: *I have some comments: The authors have been focused on the hierarchy multi-class classification which is good for developmental biology. Does the partial also perform better when there is no hierarchy in the single-cell dataset?*

Authors: Please note first that all our datasets, either artificial ones or real ones, exhibit a hierarchical organization of the labels. It is a specific feature of the task, and consequently of the real datasets, we are interested in, and we built artificial datasets with this particular feature on purpose, to study the behavior of various methods in a controlled environment.

Second, most of the results that we report have been obtained in the partially labeled setting with flat models, meaning the hierarchical organization of the labels is not exploited by the models. There is no reason why the methods proposed here would not work with datasets whose labels are not hierarchically organized.

Finally, the hierarchical organization of the labels may indeed be used with benefit. We discuss the benefit one can gain from taking into account the hierarchical organization of the labels in the **Results** section when we comment the experimental results in Fig. 8. We write in a paragraph untitled **Incorpo-**

rating hierarchy improves performance in the section (lines 650 - 657 in the main manuscript):
"... For the artificial datasets, we found that the hierarchical prototype-based performs equally well as flat-PB, and for both SVM and kNN, flat models were slightly better than hierarchical ones. We will now focus only on the experimental datasets. In the supervised setting, we find that hierarchical models perform with similar performance as Prototype-Based non-hierarchical models, slightly underperform for SVM-type models, and similarly for kNN models. However, in the partial label setting, hierarchical methods stand out and greatly improve performance."

Reviewer #1: *I appreciate that the authors have detailed their method section. But they should keep the method concise in the manuscript but can keep the elaborated methods in the supplementary.*

Authors: We followed the suggestion of Reviewer 1 to reformulate the methods more concisely. To achieve this, we did two main changes.

First, we formalized the partially labeled setting in general (with or without the hierarchical structure of the labels) and we proposed two algorithms to solve it, which are generic and may be used with various classifiers. We also provide a brief description of all the methods that we will investigate and compare. This new presentation can be found in the **Methods** section (Note that, as this section has been fully rewritten, we did not make the changes appear in the **Diff.pdf** file to ease the identification of the differences).

Second, we moved all non-trivial details on the implementation of the methods to the supplementary file **S1 Text**. This supplementary material file includes most of what was written in the **Methods** section in the previous submission.

Reviewer #1: *In the figures, the brown square is not properly visible for the best performing method. Please use a different color.*

Authors: We changed the color code based on the recommendation for publication found in [1] and applied it to ease the reading of the figures. To improve the reading again, we now report the accuracy performances in percentage, as indicated in the caption of the Figures.

Reviewer #1: *PB, SVM, and Knn are good methods. I would suggest authors use Tree based methods also like Random Forest, XGBoost, and LGBM. I think it will improve the accuracy. Also please try multiclass logistic regression.*

Authors: We thank Reviewer # 1 for this relevant comment. We considered three new models in the paper, Logistic Regression (LR) and two well-known and state-of-the-art ensemble (tree-based) methods, namely Random Forest (RF) and Extreme Gradient Boosting (XGBM). As the learning strategy that we proposed for partially labeled settings is not well suited for learning ensemble

methods we propose two versions of our algorithms, one that can be used for models whose optimization may be performed with iterative optimization like gradient descent (our original algorithm) and a variant that may be used for any other classification models (like RF, XGBM and kernel SVM). The two algorithms are detailed and motivated in the new section **Methods**.

We included the three new models in our experimental study and updated the **Results** section accordingly. Note that the learning algorithm for ensemble methods is computationally expensive, making its use prohibitive on large datasets. Hence while we study the behavior of the Logistic Regression model on all datasets, we report results of ensemble methods on the smaller (i.e. the real) datasets only.

Of course, we updated our comments and discussion on the experimental results based on these new results (see the **Diff.pdf** file that lists every single change). Yet the main messages still hold. And, more specifically to comment on the newly added baselines, the LR model most often performs similarly to the Prototype-based linear method and is outperformed by linear SVM. Ensemble-based methods are most often outperformed by either SVM or prototype-based methods on partially labeled data.

Reviewer #1: *It is good that the author has used both regular SVM and kernel SVM. I am a bit surprised that K-SVM has less F1 score than the regular SVM in single cell annotation. it could be due to the hyperparameter for K-SVM is not properly optimized, since in single cell data is non-linear. The same partial annotation works well for non-linear prototype based. Please explain.*

We thank Reviewer #1 for pointing out the difference in accuracy between regular and kernel SVM.

We decided to investigate deeper this surprising difference of performance. To start with, we remind that the non-linear SVM whose results are reported in the original submission, and this one too, are based on an approximation of the kernel. It is a well-known method whose implementation is provided in the state-of-the-art *scikit-learn* machine learning package. The advantages of such an implementation are that it enables dealing with large datasets (as artificial datasets are) since the training in partially labeled settings may be performed with the learning algorithm that we proposed in the original submission, which is computationally efficient.

We performed sanity check experiments to assess the reliability of the kernel approximation by comparing two non-linear SVM, one using true kernels while the other one relies on an approximated kernel. We first performed comparative experiments in a supervised setting and realized that the gridsearch that we used was too limited to always get good results with the kernel approximation. Using a larger gridsearch enabled us to get similar results with the two implementations. We decided to run again all the experiences for the non-linear SVM using the approximated kernel and to replace all previous results for the non-linear SVM model by the new ones. We did not include the results of SVM with a true kernel in the main paper as this implementation does not scale to

large (artificial) datasets. Yet, we provide an extensive experimental study on the implementation of non-linear SVM for our tasks in the supplementary file **S2 Text**.

At the end we now report better results for non-linear SVM, especially for the *Myeloid Progenitor* dataset, where the results in our first submission were disappointing, while the results were only slightly improved on all other datasets.

Reviewer #2: *I would like to see a comparison between the performance of your program and some others because I find it very useful to see if the program works better than others.*

We thank reviewer #2 for his useful comment. We would like first to mention the fact that partial label learning is still an uncommon machine learning task in the single-cell computational biology community. As a consequence, there is currently no implementation of a solution to the partial label learning problem for single-cell transcriptomics data available. Particularly, the main libraries like scverse [2] and seurat[3, 4], do not include any package related to learning with partially labeled data.

Next, as required by both Reviewer #2 and Reviewer #1, it is relevant to extend the scope of baseline methods to be compared, which we did by investigating, in addition to the initial SVM, Prototype-Based and kNN approaches, the multi-class logistic regression and two well known tree-based methods (Random Forest and Extreme Gradient Boosting). See answer to Reviewer #1's comment on this topic.

Finally, we hope that we have answered all the questions and would like to thank again the reviewers for their helpful comments, which helped us to improve the quality of the manuscript.

References

[1] Fabio Crameri, Grace E Shephard, and Philip J Heron. "The misuse of colour in science communication". In: *Nature communications* 11.1 (2020), p. 5444.

[2] Isaac Virshup et al. "The scverse project provides a computational ecosystem for single-cell omics data analysis". In: *Nature biotechnology* (2023), pp. 1–3.

[3] Yuhan Hao et al. "Integrated analysis of multimodal single-cell data". In: *Cell* 184.13 (2021), pp. 3573–3587.

[4] Yuhan Hao et al. "Dictionary learning for integrative, multimodal and scalable single-cell analysis". In: *Nature Biotechnology* (2023), pp. 1–12.
